# The Definition of the Term “Orthogeriatric Infection” for Periprosthetic Joint Infections

**DOI:** 10.1177/21514593221111649

**Published:** 2022-06-30

**Authors:** Nike Walter, Markus Rupp, Susanne Bärtl, Claus Uecker, Volker Alt

**Affiliations:** 1 Department of Trauma Surgery, 39070 University Medical Center Regensburg, Regensburg, Germany

**Keywords:** periprosthetic joint infection, orthogeriatric infection, multimorbidity, Barthel index, multidisciplinary treatment

## Abstract

**Introduction:**

In the background of the aging population, an increase of geriatric patients with specific age-related co-morbidities has already been seen over the years for proximal femur fractures in orthopaedic surgery as well as other medical disciplines. However, the geriatric aspect has not been well recognized in periprosthetic joint infection (PJI) patients so far. Therefore, this paper seeks to provide an overview on the co-morbidities of PJI patients with respect to the definition of geriatric patients.

**Material and methods:**

In this single-center retrospective study, patients treated between 2007 and 2020 for PJI were included (n = 255). Patients were defined as geriatric according to the consensus definition criteria of the Federal Working Group of Clinical Geriatric Facilities e.V., the German Society for Geriatrics e.V. and the German Society for Gerontology and Geriatrics e.V. based on age (≤70 years), geriatric multimorbidity and the Barthel index (≤30).

**Results:**

Applying the criteria defined 184 of the 255 (72.2%) PJI patients as geriatric infection patients. Regarding geriatric comorbidity, incontinence was most prevalent (38.1%), followed by immobility (25.6%). Comparing the geriatric infection patients with those classified as non-geriatric (n = 71**)** revealed that geriatric patients had a longer hospital stay and spent more days in the intensive care unit (ICU). Also, the amputation rate and the 5-year mortality rate was significantly increased (n = 15, 8.2% vs n = 1, 1.4%, *P* = .007 and n = 24, 13.0% vs n = 5, 7.0%, *P* = .005). The Barthel index showed a significant correlation with mortality (*r* = −.22, *P* = .011).

**Discussion:**

We propose to use the term orthogeriatric infection patients in those cases in order to focus treatment not only on the orthopaedic infections but also on the important geriatric aspects.

**Conclusion:**

The inclusion of geriatric physicians into the multidisciplinary team approach for PJI patients might be beneficial.

## Introduction

The term “geriatric orthopaedics” was firstly coined in 1974 by Michael Davis, who ran an orthogeriatric service with Bob Irvine in the Hasting Clinics in the UK.^1^ Due to the aging population, the field of geriatric orthopedics gained importance and has been grown in recent years.^2^ However, the focus has been primarily set on fragility fractures and the specific needs of the elderly requiring a comprehensive approach to care.^2,3^ For instance, it has been shown that elderly patients with fragility fractures benefit from a multidisciplinary treatment including geriatric assessment.^4-6^ Further, treating patients undergoing elective surgery aged 65 years with a team including a consultant geriatrician, nurse specialist in older people, occupational therapist, physiotherapist and social worker was reported to reduce postsurgical complications and length of hospital stay.^7^ Nevertheless, besides osteoporosis, also the prevalence of other musculoskeletal diseases increases with age such as osteoarthritis, which has been ranked as the 11^th^ highest contributor to global disability in 2010.^8^ For end stage osteoarthritis, joint replacement as a life-enhancing procedure is often the therapy choice. It was estimated that 80% of total hip arthroplasty (THA) and 96% of total knee arthroplasty (TKA) surgeries are due to osteoarthritis.^9^ In Germany, primary total knee or hip arthroplasty is among the most common procedures, with an increase in the number of surgeries of up to 45% predicted for the year 2040.^10^ Periprosthetic joint infection (PJI) is a dreaded complication in orthopaedics and trauma surgery with an incidence of 24/1 000 000 inhabitants in Germany.^11^ Hence, an increasing percentage of older patients with bone and joint infection is expectable.

Whereas there is no major differences in the pathogenesis and etiology of PJI between the adult and the geriatric population is evidenced,^12^ several studies suggest that elderly patients have a higher risk of adverse outcomes after revision total joint arthroplasty.^13,14^ Hereby, the functional status of the patients plays a major role^15^ and comorbidities such as congestive heart failure, chronic pulmonary disease, preoperative anaemia, diabetes and depression increase the risk for a subsequent infection.^16^ Also, lower success rates after 2-stage exchange arthroplasty have been reported in immunocompromised hosts^17^ as well as an increased PJI-related mortality rate in older patients.^18^

Therefore, we suggest expanding the current field of orthogeriatrics to include PJI as host optimisation plays a key role in the treatment of this specific patient population and the incorporation of comprehensive geriatric assessment could reduce complication rates, and improve the outcome and patients’ quality of life. For this purpose, it was aimed at providing an overview of the current situation, classifying PJI patients treated in our department as geriatric infection patients by age, Barthel index and comorbidities to evaluate the value of the term “orthogeriatric infection” in this context.

## Material and Methods

A single-centric retrospective cohort study of patients treated for PJI was conducted at a German level 1 trauma center. The inclusion period was defined from January 1, 2007 to December 31, 2020. Eligible patients included 18 years or older were screened by international classification of disease 10 (ICD-10) diagnosis codes (T84.5 infection and inflammatory reaction due to internal joint prosthesis). Afterwards, patients’ medical charts, surgery protocols, laboratory findings as well as microbiology and histopathology reports were screened. Inclusion criteria was a confirmed PJI. PJI was considered confirmed if at least one of the following criteria was met according to the EBJIS consensus for the diagnosis of PJI^19^ (1) sinus tract communicating with the joint, visualization of the prosthesis or purulence around the prosthesis, (2) synovial fluid analysis: leukocyte count ≥ 3000/μl or percentage of polymorphonuclear cells (PMN) ≥ 70%, (3) microbial growth ≥ 2 positive samples with the same microorganism (tissue and/or synovial fluid) or >50 colony forming units/ml in sonication fluid, and (4) histopathological findings defined as the presence of visible microorganisms or the presence of ≥ 5 PMN per 5 high-power fields (HPF). All patients with confirmed PJI were considered as eligible for the study and no patient was excluded. Patient characteristics (sex and age at the time of surgery) and details of infection (index joint, type of arthroplasty, and reinfection) were assessed retrospectively by reviewing electronic medical records. Outcome parameters such as recurrence of infection and mortality were assessed via telephone. Comorbidities were assessed using the Charlson Comorbidity Index (CCI).^20^ The Barthel index (BI) was used to assess functional independence in activities of daily living.^21^ Patients were defined as geriatric according to the consensus definition criteria of the Federal Working Group of Clinical Geriatric Facilities e.V., the German Society for Geriatrics e.V. and the German Society for Gerontology and Geriatrics e.V.^22^ Here, a geriatric patient is defined either by age (≥70 years) or by geriatric comorbidity. Geriatric comorbidity is given in cases where at least 2 of the feature complexes shown in Table 1 are present.^22^ Descriptive data analysis was performed using the IBM SPSS Statistics software (version 24.0, IBM Corp, Armonk, USA). Frequencies were expressed as numbers and percentages. Continuous parameters were presented as means *±* standard deviation (SD) and compared by Student’s t-test. Chi-square test was used for comparison of categorical variables. To determine the relationship between age, Barthel index and the number of comorbidities Pearson’s correlation was applied after determining that the distribution was appropriate for parametric testing by the Shapiro-Wilk test. For all tests, *P*-values ≤ .05 were considered statistically significant.

**Table 1.** Items of the Barthel index.^13^

Activity	Scores (Total 0-100)
**Feeding**	0 = unable 5 = needs help cutting, spreading butter, etc., or requires modified diet 10 = independent
**Bathing**	0 = dependent 5 = independent
**Grooming**	0 = needs help with personal care 5 = independent face/hair/teeth/shaving
**Dressing**	0 = dependent 5 = needs help but can do about half unaided 10 = independent
**Bowels**	0 = incontinent 5 = occasional accident 10 = continent
**Bladder**	0 = incontinent 5 = occasional accident 10 = continent
**Toilet use**	0 = dependent 5 = needs some help but can do something alone 10 = independent
**Transfers (bed to chair and back)**	0 = unable, no sitting balance 5 = major help 10 = independent
**Mobility (on level surfaces)**	0 = immobile or <50 yards 5 = wheelchair independent, including corners, >50 yards 10 = walks with help of 1 person > 50 yards 15 = independent >50 yards
**Stairs**	0 = unable 5 = needs help 10 = independent

## Results

**Figure 1.**
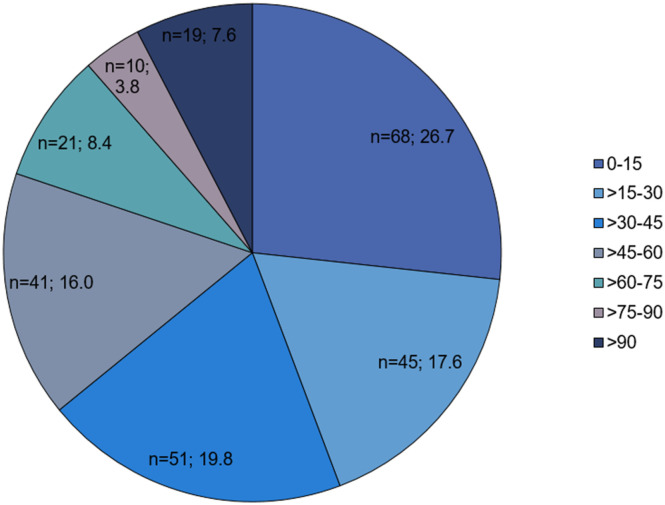
Distribution of the Barthel-index.

In total, 255 patients were identified (56.1% males) with PJI of the shoulder (7.0%), the hip (45.5%) and the knee (47.5%). The mean age was 70.7 ± 10.7 years [36-91], whereby 49 patients (19.2%) were older than 80 years and 154 patients (60.4%) were older than 70 years. The mean CCI was 2.5 ± 2.3 (range: 0-11). On average patients were hospitalized for 42.6 ± 34.8 days (range: 12-208 days). The Barthel index is a toll to assess daily living activities and mobility, such as urinal or fecal incontinence, help needed for feeding and walking etc. with a total score between 0 and 100 representing severely impaired and normal daily activities, respectively. The mean Barthel index was 40.2 ± 26.8, whereby n = 68 (26.7%) patients had a Barthel index in the range of 0-15 (Figure 1). A Barthel index ≤30 was recorded in 44.3% (n = 113) of the patients.

**Figure 2.**
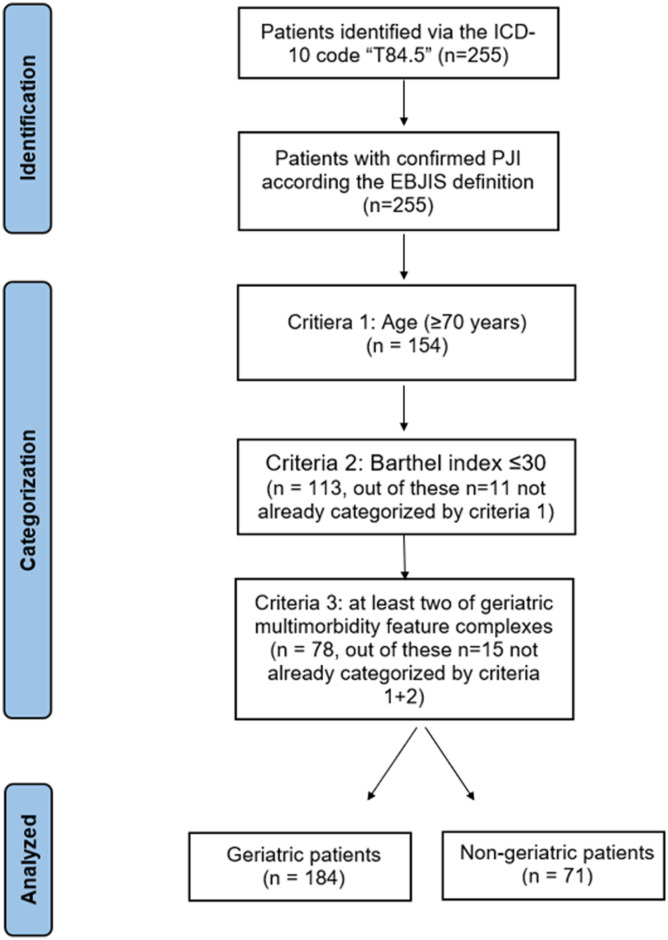
Schematic overview of the categorization process.

The analysis of comorbidities showed that n = 47 patients (18.4%) were diagnosed with 1 feature complex according to the geriatric classification. Additionally, n = 78 patients (30.6%) were diagnosed with 2 or more feature complexes for geriatric comorbidity (mean 2.7 ± .9, range 2-5). Out of these, incontinence was most prevalent (38.1%), followed by immobility (25.6%) and high complication risk (12.5%) defined as the occurrence of complications after the medical procedure, dialysis requirement, absolute arrhythmia in atrial fibrillation or the presence of artificial orifices^22^ (Table 2). Taken together, applying the criteria age ≥ 70 years, Barthel index <30, and at least 2 diagnosed multimorbidity feature complexes, yielded in n = 184 (72.2%) geriatric infection patients. Hence, n = 30 patients aged under 70 years were identified as geriatric (Figure 2). The Barthel index showed a negative correlation with age (*r* = −.31, *P*<.001). No significant correlation between the number of diagnoses out of the multimorbidity feature complexes and the age (*r* = −.02, *P* = .837) as well as the Barthel index (*r* = −.23, *P* = .136) was found.

**Table 2.** Distribution of the Geriatric Multimorbidity.

Multimorbidity Feature Complex	Number of Patients
**Immobility**	n = 62 (24.3%)
**Tendency to fall and vertigo**	n = 9 (3.5%)
**Cognitive deficits**	n = 23 (9.0%)
**Incontinence**	n = 98 (38.4%)
**Decubital ulcers**	n = 6 (2.4%)
**Malnutrition and undernourishment**	n = 7 (2.7%)
**Depression, anxiety disorder**	n = 9 (3.5%)
**Pain**	n = 2 (.8%)
**Medication problems**	n = 6 (2.4%)
**High complication risk**	n = 33 (12.9%)

Comparing the geriatric infection patients with those classified as non-geriatric revealed that a higher percentage of geriatric patients were female (n = 89, 48.3% vs n = 23, 32.4%, *P* = .021). Further, geriatric patients had a longer stay in the hospital and spent more days in the intensive care unit (ICU). Also, the amputation rate was significantly increased in geriatric PJI patients (n = 15, 8.2% vs n = 1, 1.4%, *P* = .007). Additionally, the 5-year mortality rate based on infection-related causes such as sepsis was higher in the geriatric population (n = 24, 13.0% vs n = 5, 7.0%, *P* = .005) (Table 3). The Barthel index showed a significant correlation with mortality (*r* = −.22, *P* = .011).

**Table 3.** Patient Characteristics and Outcomes Divided in the Geriatric and Non-Geriatric Population. *P ≤ .05.

	Geriatric	Non-geriatric	*P*-value
Patients	n = 184	n = 71	
Gender			.021*
**Male**	n = 95 (51.6%)	n = 48 (67.6%)	
**Female**	n = 89 (48.3%)	n = 23 (32.4%)	
Age	75.0 ± 8.0 years	59.6 ± 8.2 years	<.001*
Anatomical localization			.416
**Shoulder**	n = 11 (6.0%)	n = 18 (7.0%)	
**Femur**	n = 87 (47.3%)	n = 116 (45.5%)	
**Knee**	n = 86 (46.7%)	n =121 (47.5%)	
Treatment			.767
**DAIR**	n = 45 (24.5%)	n = 21 (29.6%)	
**Antibiotic suppression**	n = 5 (2.7%)	n = 1 (1.4%)	
**One-stage exchange**	n = 57 (31.0%)	n = 19 (26.8%)	
**Two-stage exchange**	n = 44 (23.9%)	n = 19 (26.8%)	
**Girdlestone**	n = 7 (3.8%)	n = 1 (1.4%)	
**Arthrodesis**	n = 11 (6.0%)	n = 9 (12.7%)	
**Amputation**	n =15 (8.2%)	n = 1 (1.4%)	.007*
Difficult-to-treat pathogen	n = 14 (7.6%)	n = 8 (11.3%)	.053
CCI	2.7 ± 2.3 [0-11]	2.1 ± 2.2 [0-11]	.103
Barthel index	31.7 ± 24.3	60.9 ± 20.8	<.001*
Length of hospital stay	45.4 ± 28.5 days	35.5.4 ± 29.1 days	.043*
Length of ICU stay	7.5 ± 5.3 days	3.1 ± 1.6 days	.032*
Recurrence of infection	n = 27 (14.7%)	n = 22 (31.0%)	.003*
Revision rate	1.3 ± 1.8	1.8 ± 2.2	.107
Infection-related 5-year mortality	n = 24 (13.0%)	n = 5 (7.0%)	.005*

DAIR = debridement, antibiotics and implant retention, CCI = Charlson Comorbidity Index, ICU = intensive care unit.

## Discussion

The purpose of this study was to introduce a definition of the term “orthogeriatric infection”. Here, 72.2% of all PJI patients were categorized as geriatric infection patients. Based on the consensus definition criteria of the Federal Working Group of Clinical Geriatric Facilities e.V., the German Society for Geriatrics e.V. and the German Society for Gerontology and Geriatrics e.V.,^22^ age of ≥70 years was chosen for the classification of patients. However, the heterogeneity of the definition of elderly age in orthopedic research has been highlighted.^23^ In a meta-analysis including 80 studies and 271 470 patients it was shown that 95% of the studies defined elderly age solely based on chronology with a range from 50 to 80 years, whereby 65 years was most commonly used as a cutoff (47.5%). Therefore, including a fraility index is deemed beneficial for an improved approach in orthopaedic research focusing on the elderly.^23^ Here, the Barthel index was applied, which has a sufficient structural validity, reliability, and interpretability and depicts a valuable tool to assess the ability to perform daily activities, especially in geriatric patients.^24^ The Barthel index was shown to have a strong and independent association with mortality in geriatric patients.^25^ Especially in patients with hip fractures, a lower Barthel index was associated with increased mortality serving as an independent risk factor,^26,27^ which is in line with our findings of a significant association between the Barthel index and the mortality rate in the present cohort. Also, lower Barthel indices were determined in patients with fracture-related infection of the hip compared with a matched control group without infection.^28^ A recent study conducted in Spain collected the Barthel scores in 2 nursing homes before and after an infection with Covid-19. In their cohort (mean age 85.9 ± 6.42, 34 male/34 female) the post Covid Barthel total score was 52.30 ± 27.22 (44.56-60.04), which was higher than in the cohort analyzed here (40.2 ± 26.8), emphasizing the burden of PJI.^29^ Further, the geriatric multimorbidity was considered. The results showed that patients were diagnosed with 2.7 ± .9 multimorbidity feature complexes on average and that incontinence (38.1%) and immobility (25.6%) were the most prevalent. Also here, heterogeneity in the literature can be noted. For instance, a recent meta-analysis reported a prevalence of delirium among orthopedic surgery patients between 4.5% and 41.2%.^30^ Vetrano et al. investigated geriatric syndromes in 6903 participants presented with an average of 2.0 geriatric symptoms with pain (48%), urinary incontinence (47%) and falls (33%) being the most prevalent.^31^ Another study conducted in France analysed geriatric symptoms in people aged 75 years and older reported that the most frequent geriatric syndromes were polypharmacy (50.6%, 95%CI = 46.7-54.5) and falls (43.1%, 95%CI = 38.4-46.1). Whereas here no significant association between the type of feature complex (e.g immobility and incontinence) was found, in general, comorbidity of the patients plays an essential role in the treatment and prognosis for PJI. Therefore, also considering the higher amputation rate reported in this study in the geriatric population, alternatives therapy strategies for elderly patients with PJI who would not benefit or survive surgical treatment have to be addressed.^32^ For instance, Prendki et al. recorded that 60% of (n = 38) patients in the age range 80-95 years remained event-free during 2 years of prolonged suppressive antibiotic therapy.,^33^ Other authors have shown a high success rate (84% of n = 26 patients) for prolonged antibiotic suppression therapy.^34^ Additionally, a significantly higher infection-related mortality rate was found in the geriatric population, which is in line with other findings showing that PJI-related death is more common in older patients (6.5% vs .8%, *P* < .05).^18^

Thus, to achieve the best outcome for the patient, interdisciplinary approaches and early involvement of multidisciplinary teams are deemed important. For instance, it was demonstrated that patients treated for PJI of the hip had a shorter in-hospital stay, reduced numbers of surgeries and less antibiotics when discussed with a multidisciplinary team.^35^ In addition, Bauer et al. analyzed files of patients treated for bone and joint infection before and after the implementation of a multidisciplinary staff meeting, reporting optimized adaptation of antibiotic therapy.^36^ A similar approach with valuable clinical experiences is reported by Carlson and colleagues with a collaboration between infectious disease clinicians and orthopaedic arthroplasty surgeons to optimize PJI treatment.^37^ Recently, also raising numbers of psychological comorbidities over the years have been shown for PJI patients, whereby the authors concluded that interdisciplinary collaboration is warranted and a psychologists should be included in the management.^11^ In light of the presented findings, routine geriatric screening assessment should be considered. Further, the high number of geriatric infection patients (72.2%) emphasizes that geriatric physicians should be included as part of the PJI treatment team.

The study shows limitations. The first depicts its retrospective design, which did not allow to present longitudinal changes of the Barthel index. Further, correct coding of the comorbidities was assumed, although it is not fully assured.

In conclusion, to prepare for the changing demographics among bone and joint infection patients, geriatric infection-oriented trainings should be provided to improve patient’s quality of life, surgical outcomes, and reduce healthcare cost. Interdisciplinary collaborations among orthopaedic surgeons, geriatric physicians, physiotherapists and psychologists should be strengthened. Decision makers in healthcare systems should support future efforts to enhance not only the quantity but quality of life of patients with geriatric bone and joint infections.
